# Recorded fracture liaison service plus discharge zoledronic acid prescription and 730-day refracture risk after surgically treated vertebral fracture in older adults: a target-trial-informed discharge landmark analysis

**DOI:** 10.3389/fmed.2026.1972395

**Published:** 2026-10-14

**Authors:** Ke Gao, Gang Zhang, Haoran Lu, Xiangyang Liu, Yubing Zhang

**Affiliations:** 1 Department of Spine Surgery, Anhui No. 2 Provincial People’s Hospital, Hefei, Anhui, China; 2 The First Affiliated Hospital of Anhui University of Science and Technology, Huainan, Anhui, China; 3 Department of Clinical Medicine, Wannan Medical University, Wuhu, Anhui, China

**Keywords:** competing risk, fracture liaison service, landmark analysis, older adults, secondary fracture prevention, vertebral fracture, zoledronic acid

## Abstract

**Background:**

Hospitalization for vertebral fracture offers an opportunity to connect acute care with secondary fracture prevention. We examined whether recorded fracture liaison service (FLS) use plus a positive discharge zoledronic acid (ZOL) prescription/dispensing field was associated with lower postdischarge refracture risk.

**Methods:**

Using a public fracture registry from Kunshan, China, we included patients aged 65 years or older admitted between 2019 and 2021 with a surgically treated thoracic or lumbar vertebral fracture. Eligible patients were alive and refracture-free at discharge, with discharge defining the landmark time zero. We compared recorded FLS plus a positive discharge ZOL prescription (pathway) against neither a recorded FLS nor a discharge ZOL/alendronate (comparator). Fifty-fold multiple imputation and ridge propensity score overlap weighting addressed missing measured covariates and group differences. Weighted Aalen–Johansen estimation treated death as a competing event. Confidence intervals (CIs) were derived from 1,000 patient-level bootstrap replicates.

**Results:**

The cohort included 585 patients (266 pathway; 319 comparator), with 37 refractures and 26 deaths before refracture. The maximum weighted absolute standardized mean difference was 0.046 for fitted propensity terms and 0.087 for the common clinical panel across imputations. Estimated 730-day refracture risks were 2.40% (95% CI, 0.76%–4.31%) and 7.02% (95% CI, 4.12%–10.29%), respectively. The risk difference was −4.63 (95% CI, −8.38 to −1.24) percentage points and the risk ratio was 0.34 (95% CI, 0.10–0.76). Reduced and alternative propensity specifications yielded similar associations, although some left greater residual imbalance. The extended 2019–2022 analysis (*n* = 770) at 365 days was less conclusive [risk difference, −1.24 (95% CI, −3.62 to 1.07) percentage points].

**Conclusions:**

Among older surgically treated vertebral fracture inpatients, recorded FLS plus positive discharge ZOL prescription was associated with lower estimated 730-day refracture incidence. This pathway-level observational association does not establish the independent effects of FLS or ZOL or confirm drug administration. Limited events and unmeasured treatment selection factors warrant cautious interpretation.

## Introduction

Osteoporotic vertebral fracture is a sentinel event in later life: The risk of another vertebral fracture rises sharply during the following year, and subsequent low-trauma fractures are associated with excess mortality (1, 2). However, many patients leave fracture care without systematic osteoporosis (OP) assessment or pharmacologic secondary prevention, despite consensus recommendations for older adults with hip or vertebral fracture (3, 4). Hospitalization, therefore, provides a time-limited opportunity to connect acute vertebral fracture care with a durable secondary prevention plan.

Fracture liaison services (FLSs) were developed to close this gap by coordinating case identification, osteoporosis assessment, treatment initiation, and follow-up across specialties and care settings (5, 6). Meta-analyses suggest that FLS models improve treatment delivery and may reduce subsequent fractures, although their organization and effectiveness vary across health systems (7, 8). Pharmacotherapy is integral to these pathways, and bisphosphonates remain central to osteoporosis treatment (9). Randomized trials have demonstrated fracture risk reduction with alendronate (ALN) and zoledronic acid (ZOL) (10, 11). Zoledronic acid may be particularly useful during care transition because its delivery does not depend on continued oral dosing, and its antifracture efficacy has been established in postmenopausal osteoporosis and after hip fracture (11, 12). Because bisphosphonate benefits accrue over time, outcomes beyond the immediate postdischarge period are clinically relevant (13). An observational implementation study showed that integrating orthopedic and FLS care with inpatient zoledronic acid increased osteoporosis treatment rates, but it did not evaluate long-term fracture outcomes (14).

Whether a recorded FLS status coupled with a discharge ZOL prescription/dispensing record is associated with lower long-term refracture risk, particularly among older surgically treated vertebral fracture inpatients, remains unknown. Addressing this gap with observational data requires explicit alignment of eligibility, pathway classification, time zero, and follow-up to limit immortal time and related design biases (15, 16). We therefore used a public longitudinal registry to conduct a target-trial-informed discharge landmark comparison of a recorded FLS indicator plus a positive discharge ZOL field versus neither recorded FLS nor a positive discharge bisphosphonate field, estimating the difference in 730-day postdischarge refracture incidence (17).

## Methods

### Study design and data source

We conducted a retrospective, target-trial-informed discharge landmark analysis using the public, de-identified Osteoporotic Fracture Registration System from the Affiliated Kunshan Hospital of Jiangsu University, China (17). The release contains one clinical record for each of 4,782 patients and includes clinical characteristics, discharge medication fields, and longitudinal fracture and mortality outcomes. The data creators linked the fracture registry to regional health and death registration systems through encrypted identifiers and verified refractures against clinical records. The Kunshan Regional Health Registration Platform (RHRP) captures inpatient and outpatient care recorded in all public hospitals in Kunshan, and linkage to the Jiangsu Provincial Death Registration System (PDRS) was complete for registered patients (100% matching in the source description). Refractures occurring outside Kunshan, in non-public institutions, or outside the linked regional systems may therefore be missed, and outcome under-ascertainment may differ by exposure group. We analyzed only the released, de-identified data and performed no additional person-level linkage. Patient selection and analysis were performed using the clinical table, with the variable definition table guiding interpretation of the released fields; the separate longitudinal antihypertensive-prescription table was not used. The data are publicly available through Figshare (18).

### Eligibility and discharge landmark pathways

The primary cohort comprised patients aged 65 years or older admitted in 2019–2021 with a thoracic (S22.0x) or lumbar (S32.0x) vertebral fracture code; valid admission–operation–discharge chronology; and survival without refracture to discharge. All released records contained an operation date and procedure code, so the study concerns the represented surgical population. The 2019–2021 period allowed every eligible discharge to reach 730 days before the refracture ascertainment cutoff date of 24 March 2024. The 730-day horizon covers the high-risk period after vertebral fracture and extends beyond the expected time required for bisphosphonate therapy to begin reducing fracture risk (1, 13). The anatomical International Classification of Diseases (ICD) phenotype does not independently establish osteoporotic etiology. We therefore audited the released ICD, recorded OP, and bone density fields; these lacked sufficient diagnostic information for an independently validated etiologic definition. Operational definitions, the discharge landmark specification, and candidate phenotype screens are provided in Supplementary Tables S1–S3. All eligible records were included without an *a priori* sample size calculation.

The pathway required recorded FLS use (FLS > 0) and a positive discharge ZOL field (ZOL > 0). The comparator required FLS = 0, ZOL = 0, and ALN = 0. The data dictionary defines ZOL as the number of boxes prescribed/dispensed at discharge, not an infusion administration log. A positive field therefore identifies a prescription/dispensing record, rather than verified infusion or a separately coded recommendation. FLS-only and bisphosphonate-only patterns were excluded from binary comparisons and described separately. Although the dictionary labels RYQzoltime as a pre-admission ZOL-use date, all 1,121 non-missing released dates fell between admission and discharge. We used this field solely to restrict a sensitivity analysis to dated ZOL records, without treating it as proof of administration.

The FLS field denotes recorded service use but has no service date. We treated it as an index care attribute for discharge landmark classification; its occurrence before discharge could not be independently verified. Discharge aligns follow-up with the discharge medication record, but does not remove uncertainty about FLS timing. This is a comparison of recorded states among discharge survivors, not randomized assignment or verified initiation of FLS or intravenous ZOL.

The release does not document an FLS-specific case identification protocol, falls assessment, patient education, scheduled follow-up, or coordination with primary care. Osteoporosis, bone density, laboratory and medication fields are available, but one cannot establish which assessments or treatments were delivered through FLS. Available registry fields do not establish component-level FLS service processes (Supplementary Tables S1, S2).

### Outcome and covariates

Follow-up began at discharge. The primary outcome was the first recorded hospitalized refracture within 730 days; death before refracture was treated as a competing risk. The source registry describes subsequent osteoporotic fractures involving the wrist, proximal humerus, hip, vertebrae, or rib, but the release does not provide a complete outcome ICD algorithm or an independent adjudication sample. Refracture and survival intervals were checked against source dates and were already discharge-based, so length of stay was not subtracted. Refracture was assigned when it occurred within the horizon before or on the recorded death date; death was assigned when it occurred first. Event-free patients in the mature primary cohort were observed through day 730, with no earlier administrative censoring.

The primary propensity model included age, sex, admission year and quarter, thoracic versus lumbar fracture, procedure family, and Charlson Comorbidity Index (CCI); it also included recorded histories of hypertension, diabetes, heart disease, pulmonary disease, kidney disease, brain/cerebrovascular disease and malignancy; smoking; alcohol consumption; systolic and diastolic blood pressure; body mass index (BMI); hemoglobin; albumin; creatinine; urea nitrogen; calcium; neutrophils; and lymphocytes. Covariates represented clinical characteristics potentially related to pathway selection, refracture or competing death. CCI summarizes overall comorbidity burden; patients with the same score may have different disease combinations. The seven broad history fields are not the complete set of Charlson components. Kidney disease history records a diagnosis, whereas creatinine and urea nitrogen provide related but distinct continuous biochemical information. These measures do not establish renal eligibility for ZOL. CCI was used as recorded, with age modeled separately. Procedure family distinguished vertebral augmentation from fixation/fusion. These were predischarge clinical variables, not uniformly verified admission baseline measurements. American Society of Anesthesiologists (ASA) class was excluded from the primary model and added in a sensitivity analysis. Supplementary Table S4 gives the exact encoded terms for the primary and alternative clinical specifications.

### Statistical analysis

Missing continuous covariates were multiply imputed using chained equations with predictive mean matching (50 imputations; 5 donors; 10 initial cycles). Predictors included clinical covariates, exposure, event indicators, log-transformed follow-up time, and exposure–event interactions (Supplementary Tables S4, S5). Within each complete dataset, we fitted L2-regularized logistic propensity scores (PS) (C = 1.0; 29 non-intercept primary terms), with standardized non-dummy terms and one-hot calendar coding. Overlap weights were 1 − PS for pathway patients and PS for comparators (19, 20). We evaluated score distributions, effective sample size (ESS), matrix rank, and variance inflation factors (VIFs). Balance was assessed for fitted terms and the common clinical panel using absolute standardized mean differences (SMDs), with 0.10 as a conventional threshold (21). Medians and maxima across imputations are reported separately (Supplementary Tables S6, S7). Group-specific Aalen–Johansen cumulative incidences treated death as competing risk (22). Risks were averaged across imputations before calculating the risk difference (RD; pathway minus comparator, in percentage points) and risk ratio (RR). Each of 1,000 patient-level bootstrap samples repeated the sequence of restriction, imputation, propensity fitting, and outcome estimation; 95% confidence intervals (CI) used the 2.5th and 97.5th percentiles of the pooled patient sample estimates. Unweighted risks were descriptive. Confidence intervals for sensitivity analyses were exploratory and not adjusted for multiple comparisons. Additional details on propensity score specifications, missing data handling, and design diagnostics are provided in Supplementary Tables S4–S9.

Sensitivity analyses evaluated disease domain and CCI-based representations, an 11-term clinical-core PS, separate CCI/urea nitrogen omissions, median imputation with independent missingness indicators, complete cases, dated ZOL records, percutaneous kyphoplasty (PKP)-only procedures, common support, and ASA addition (Supplementary Tables S8, S9). The admission record reduced model retained age, sex, admission year and quarter, thoracic subtype, CCI, the seven disease histories, smoking, alcohol consumption, and systolic and diastolic blood pressure. It excluded procedure, BMI, ASA, and laboratory measurements from both PS and imputation predictors because their admission timing was uncertain; the dictionary describes blood pressure as measured at admission, but not every retained field had an independent time stamp. We therefore use “admission-record reduced” rather than “admission-certain.” Single-term CCI/urea omissions instead retained the full imputation predictor pool. Death-censored 1-minus-Kaplan–Meier net risk was supportive. The extended 2019–2022 cohort used 365 days because many later discharges could not reach 730 days. Follow-up maturity, subset event distributions, and leave-one-refracture-patient influence were descriptive. During revision, the primary missing data approach was changed from median imputation to multiple imputation (MI) after completion of the original analysis. Additional sensitivity analyses were conducted as part of the revision and were not preregistered. Analyses were performed using Python 3.14.3 with pandas 2.3.3, NumPy 2.4.4, scikit-learn 1.8.0, SciPy 1.17.1, statsmodels 0.14.6, and joblib 1.5.3, with figures generated using Matplotlib.

## Results

### Cohort construction

Among 4,782 released records, 1,393 were on patients aged 65 years or older admitted in 2019–2021. Of these, 596 had a strict S22.0x/S32.0x vertebral fracture code. No patients in this anatomical subset were excluded for invalid admission–operation–discharge chronology (*n* = 0), and all survived to discharge. One patient had a refracture by discharge, leaving 595 landmark-eligible patients. Excluding 10 FLS-only or bisphosphonate-only patterns yielded 585 patients: 266 in the recorded FLS-plus-ZOL pathway and 319 in the comparator (Figure 1). The phenotype audit identified no released M80/M84 codes; requiring OP = 1 would retain only 212 patients (130 pathway; 82 comparator) and would not independently establish fracture etiology (Supplementary Table S3).

**Figure 1:**
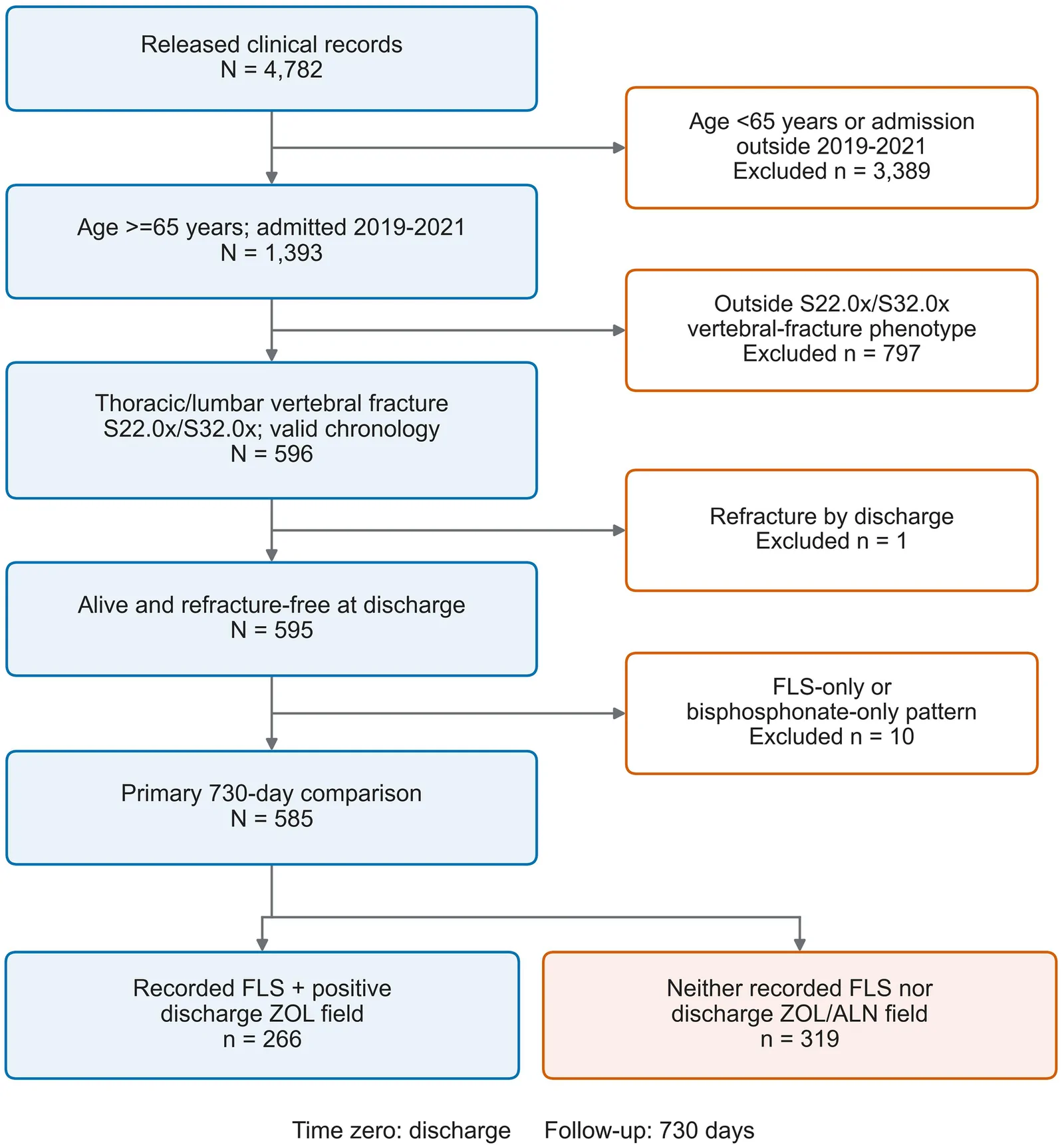
Selection of the discharge landmark cohort. Patients were aged 65 years or older and admitted in 2019–2021. Invalid chronology excluded 0 of the 596 patients with the anatomical vertebral fracture phenotype; all survived to discharge, and 1 was excluded for a refracture by discharge. Ten component-only patterns were excluded from the binary comparison. FLS, fracture liaison service; ZOL, zoledronic acid; ALN, alendronate. Positive drug fields denote discharge prescription/dispensing records.

### Baseline characteristics and design diagnostics

Before weighting, the pathway group was slightly younger, included fewer men, and had a greater proportion of admissions in 2020 than the comparator group (Table 1). Vertebral augmentation accounted for 259/266 pathway procedures (97.4%) and 301/319 comparator procedures (94.4%). Across 50 complete datasets, the primary model had 29 terms and full column rank, with a maximum VIF of 4.29 and a median total ESS of 453.2. The maximum weighted absolute SMD was 0.046 for fitted terms and 0.087 for the common clinical panel (Supplementary Figures S1, S2; Supplementary Tables S6, S7). Systolic blood pressure (SBP) and diastolic blood pressure (DBP) were each missing for the same 160 patients (27.4%): 66/266 pathway patients (24.8%) and 94/319 comparators (29.5%). BMI was missing for 67 patients (11.5%); each remaining incomplete numeric covariate was missing in no more than 12 patients (2.1%) (Supplementary Table S5).

**Table 1:** Patient characteristics and propensity score balance.

Characteristic	Pathway (*n* = 266)	Comparator (*n* = 319)	Absolute SMD before	Absolute SMD after
Age, years	72.9 (5.9)	73.7 (6.7)	0.128	0.000
Male sex	48 (18.0)	94 (29.5)	0.271	0.004
Admission in 2019	42 (15.8)	143 (44.8)	Not fitted	Not fitted
Admission in 2020	136 (51.1)	71 (22.3)	0.628	0.041
Admission in 2021	88 (33.1)	105 (32.9)	0.004	0.021
Thoracic vertebral fracture	107 (40.2)	121 (37.9)	0.047	0.004
Fixation or fusion	7 (2.6)	18 (5.6)	0.152	0.046
Charlson Comorbidity Index	0.11 (0.36)	0.17 (0.55)	0.131	0.006
Hypertension history	50 (18.8)	37 (11.6)	0.202	0.004
Diabetes history	10 (3.8)	15 (4.7)	0.047	0.001
Heart disease history	8 (3.0)	10 (3.1)	0.007	0.001
Pulmonary disease history	7 (2.6)	9 (2.8)	0.012	0.001
Kidney disease history	1 (0.4)	6 (1.9)	0.143	0.002
Brain/cerebrovascular history	9 (3.4)	8 (2.5)	0.052	0.001
Malignancy history	3 (1.1)	3 (0.9)	0.019	0.002
Smoking history	14 (5.3)	20 (6.3)	0.043	0.002
Alcohol-consumption history	10 (3.8)	21 (6.6)	0.128	0.001
Systolic blood pressure, mmHg	137.5 (18.3)	139.7 (19.3)	0.110	0.002
Diastolic blood pressure, mmHg	76.6 (11.4)	77.2 (9.6)	0.072	0.002
Body mass index, kg/m^2^	23.2 (3.2)	23.1 (3.0)	0.025	0.001
Hemoglobin, g/L	125.3 (18.1)	123.4 (19.7)	0.100	0.001
Albumin, g/L	39.9 (4.5)	39.6 (4.2)	0.075	0.002
Creatinine, μmol/L	67.7 (23.5)	71.1 (35.1)	0.109	0.003
Urea nitrogen, mmol/L	5.9 (2.3)	5.9 (2.5)	0.007	0.003
Calcium, mmol/L	2.19 (0.13)	2.20 (0.12)	0.031	0.002
Neutrophils, 10^9^/L	7.01 (3.49)	6.45 (2.80)	0.177	0.002
Lymphocytes, 10^9^/L	1.26 (0.57)	1.22 (0.54)	0.077	0.001

Values are available-case mean (SD) or *n* (%), before imputation. Before-weighting SMD is the median absolute SMD across 50 complete datasets; after-weighting SMD is the maximum absolute SMD across those datasets. Admission year 2019 is the omitted reference category, not a fitted term. Quarter indicators and the wider clinical panel are presented in Supplementary Table S6. The pathway group comprised patients with recorded FLS use and a positive discharge ZOL field, whereas the comparator group comprised patients with no recorded FLS use and neither a positive discharge ZOL field nor a positive discharge ALN field.

### Primary 730-day refracture incidence

During follow-up, 37 refractures occurred: 8 among 266 pathway patients and 29 among 319 comparator patients. Twenty-six patients died before refracture, with 12 in the pathway group and 14 in the comparator group. Unweighted cumulative refracture risks were 3.01% and 9.09%. After overlap weighting and pooling across imputations, risks were 2.40% (95% CI, 0.76%–4.31%) and 7.02% (95% CI, 4.12%–10.29%) (Figure 2 and Table 2). The RD was −4.63 (95% CI, −8.38 to −1.24) percentage points, and the RR was 0.34 (95% CI, 0.10–0.76).

**Figure 2:**
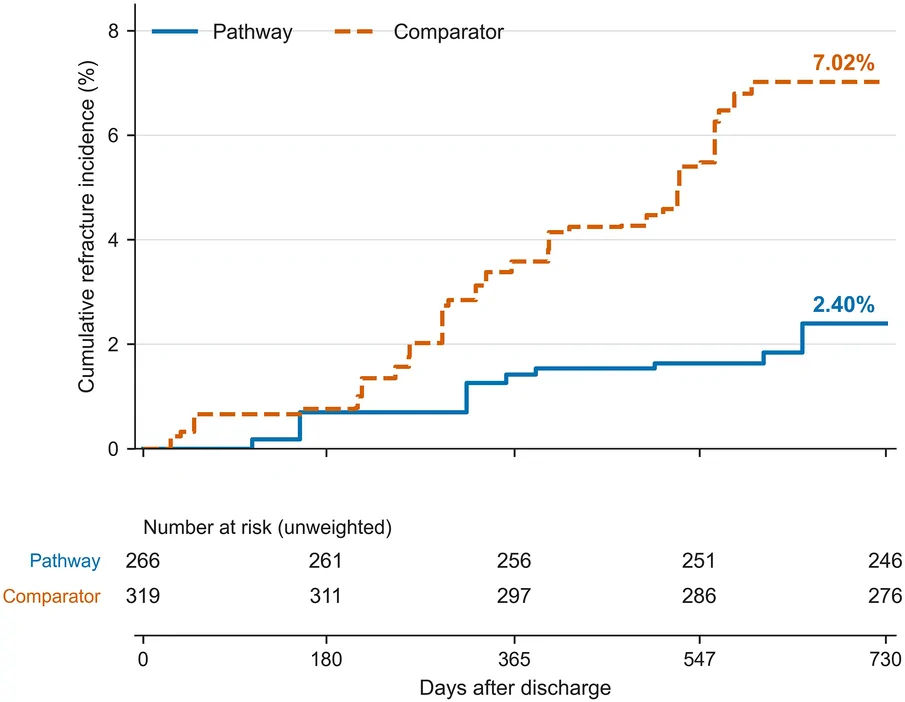
Overlap-weighted cumulative incidence of refracture after discharge. Aalen–Johansen curves show the first recorded hospitalized refracture, with death before refracture as a competing event. Curves are averaged at common time points across 50 multiply imputed datasets; each dataset has separately fitted propensity scores and overlap weights. The risk table gives unweighted event-free patients still under observation. Pointwise confidence bands were not estimated. Endpoint confidence intervals are provided in Table 2. Pathway: recorded FLS plus positive discharge ZOL; comparator: recorded neither FLS nor positive discharge ZOL/ALN.

**Table 2:** 730-day refracture incidence and pathway contrasts.

Analysis	Pathway risk, % (95% CI)	Comparator risk, % (95% CI)	RD, percentage points; RR (95% CI)
Unweighted descriptive	3.01	9.09	RD −6.08; RR 0.33
Overlap-weighted primary	2.40 (0.76 to 4.31)	7.02 (4.12 to 10.29)	RD −4.63 (−8.38 to −1.24); RR 0.34 (0.10 to 0.76)

CIF, cumulative incidence function; CI, confidence interval; PS, propensity score; RD, risk difference; RR, risk ratio.

*N* = 585 (pathway 266; comparator 319). Refractures: 8 and 29; deaths before refracture: 12 and 14, respectively. Weighted risks are Aalen–Johansen cumulative incidences averaged across 50 imputations, with death as a competing event. The RD is pathway minus comparator. Confidence intervals use 1,000 patient-level bootstrap samples with imputation and PS fitting repeated within each sample. Unweighted estimates are descriptive without inferential intervals. Rounded risks need not subtract exactly to the independently rounded RD.

### Sensitivity analyses

Model specification and restriction analyses generally yielded associations in the same direction as the primary estimate (Figure 3 and Table 3; Supplementary Table S8). The 11-term clinical-core model estimated an RD of −4.54 (95% CI, −7.75 to −0.90) percentage points. Corresponding RDs were −4.92 (95% CI, −8.61 to −1.39) after omitting CCI from the PS and −4.74 (95% CI, −8.46 to −1.34) after omitting urea nitrogen. The admission record reduced analysis estimated −4.65 (95% CI, −8.28 to −1.45) percentage points. These were sensitivity estimates, not independent tests of the pathway components.

**Figure 3:**
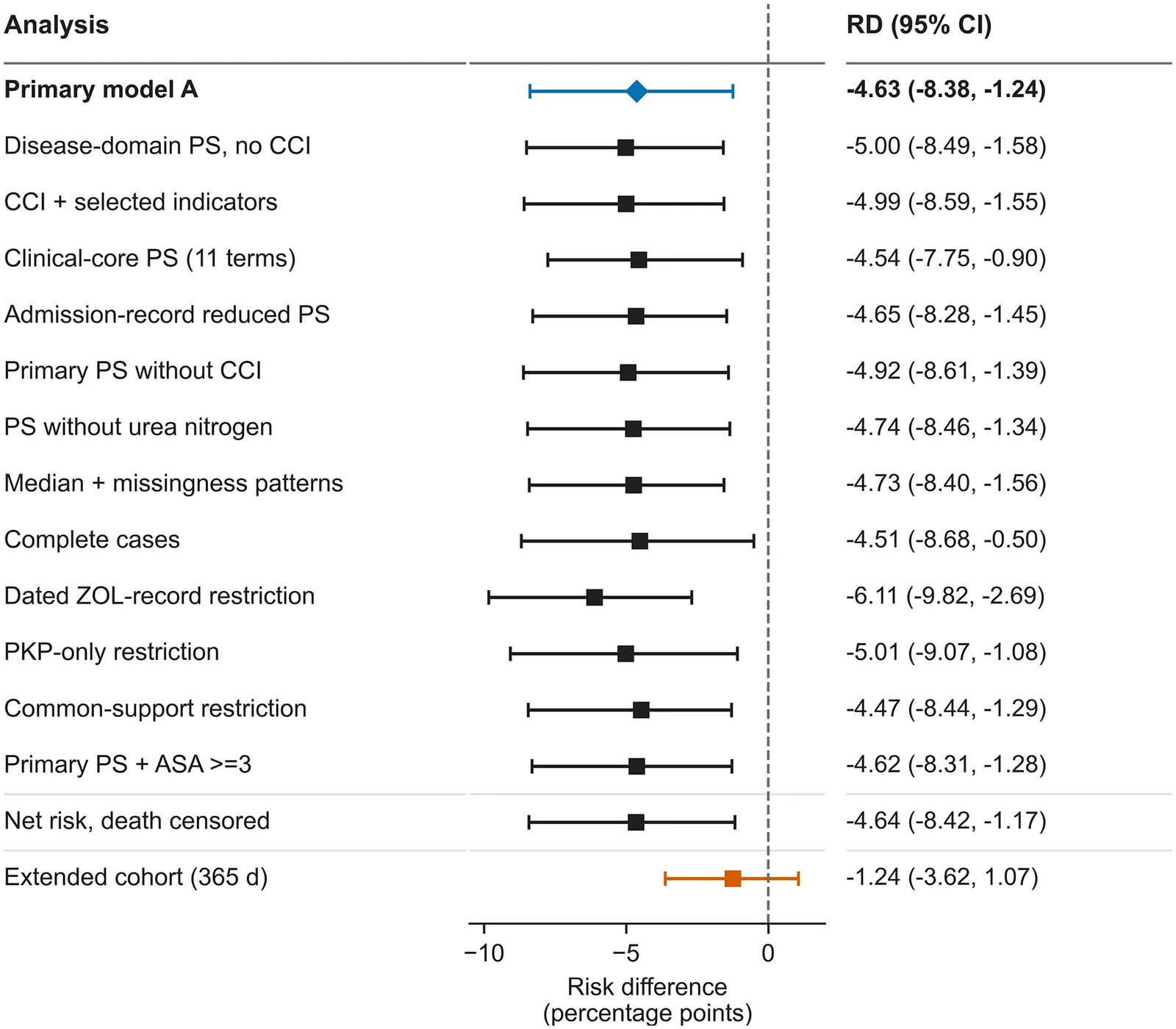
Primary and sensitivity estimates of absolute refracture risk difference. Symbols and lines show overlap-weighted RDs and percentile 95% CIs from 1,000 patient-level bootstrap samples per analysis. Negative values indicate lower estimated risk in the recorded FLS-plus-ZOL pathway. Thirteen rows estimate 730-day Aalen–Johansen incidence; death-censored net risk and the 365-day extension have different targets. Intervals are exploratory and are not adjusted for multiple comparisons. Full estimates and diagnostics are in Supplementary Tables S8, S9. CCI, Charlson Comorbidity Index; PS, propensity score; PKP, percutaneous kyphoplasty; ASA, American Society of Anesthesiologists.

**Table 3:** Selected sensitivity analyses.

Analysis	*N*	RD, percentage points (95% CI)	RR (95% CI)
Clinical-core PS (11 terms)	585	−4.54 (−7.75 to −0.90)	0.40 (0.14 to 0.82)
Admission record reduced PS	585	−4.65 (−8.28 to −1.45)	0.34 (0.11 to 0.72)
Primary PS without CCI	585	−4.92 (−8.61 to −1.39)	0.33 (0.10 to 0.72)
Primary PS without urea nitrogen	585	−4.74 (−8.46 to −1.34)	0.34 (0.11 to 0.75)
Complete cases	392	−4.51 (−8.68 to −0.50)	0.27 (0.02 to 0.85)
Dated ZOL record restriction	565	−6.11 (−9.82 to −2.69)	0.11 (0.01 to 0.33)
Common support restriction	536	−4.47 (−8.44 to −1.29)	0.35 (0.10 to 0.76)
Extended 2019–2022 (365 days)	770	−1.24 (−3.62 to 1.07)	0.59 (0.09 to 1.56)

CCI, Charlson Comorbidity Index; PS, propensity score.

All rows use 1,000 patient-level bootstraps and 730-day Aalen–Johansen risks except the 365-day extension. CCI/urea omissions change only the PS, retaining the full imputation pool.

Similar effect estimates did not imply equivalent covariate balance. Maximum weighted absolute SMDs in the common panel were 0.185 without CCI, 0.178 without urea nitrogen, and 0.322 with the 11-term clinical-core model, compared with 0.087 in the primary analysis (Supplementary Figure S3; Supplementary Table S9). The dated record restriction estimated an RD of −6.11 (95% CI, −9.82 to −2.69) percentage points; however, four of the eight pathway refractures occurred among 246 patients with a dated record and four among 20 without one (Supplementary Table S10). Leave-one-refracture-patient estimates ranged from −5.19 to −3.87 percentage points (Supplementary Figure S4). All 15 inferential analyses had 1,000 complete bootstrap samples. One common-support bootstrap draw had a rank-deficient design in at least one imputation; its finite ridge estimate was retained and independently verified.

### Extended 2019–2022 cohort and recorded component patterns

The extended 2019–2022 source cohort included 814 discharge landmark patients; excluding 14 FLS-only and 30 bisphosphonate-only patterns left 770 for binary inference. The 365-day horizon was used because follow-up at 730 days was incomplete for many 2022 discharges. The 365-day RD was −1.24 (95% CI, −3.62 to 1.07) percentage points and the RR was 0.59 (95% CI, 0.09 to 1.56). All 770 patients could potentially complete 365 days, whereas only 22/58 pathway patients and 19/127 comparators admitted in 2022 could potentially complete 730 days (Supplementary Figure S5; Supplementary Table S11). There were 137 event-free early administrative censoring events at the longer horizon. Because the extension changed both the calendar cohort and follow-up duration, it is not a simple time course comparison with the primary result.

The extended source cohort contained 446 patients with neither component, 14 with FLS only, 30 with bisphosphonate only, and 324 with recorded FLS plus positive ZOL (Supplementary Table S12). All 324 pathway patients had positive ZOL fields and five also had positive ALN. Supportive medication fields were positive for calcium in 338 patients, vitamin D in 673, and calcitonin in 213 (Supplementary Table S13). These overlapping recorded patterns were descriptive and did not support independent component-effect estimation.

## Discussion

In this discharge landmark analysis of older surgically treated vertebral fracture inpatients, recorded FLS plus a positive discharge ZOL prescription/dispensing field was associated with an estimated 4.63-percentage-point lower 730-day refracture incidence than the comparator; the RR was 0.34. Alternative propensity specifications and clinical restrictions yielded associations in the same direction, although simplified models left larger residual differences in the common clinical panel. The extended 2019–2022 estimate at 365 days was smaller and its interval included the null. Because that analysis changed the calendar cohort and horizon, it does not establish when a pathway association might emerge.

The primary finding is broadly consistent with the mixed observational literature on FLS outcomes. A systematic review and meta-analysis associated FLS participation with fewer subsequent fractures, and a large Swedish implementation study similarly reported lower recurrent fracture risk after FLS introduction (23, 24). Controlled cohort studies by Nakayama et al. and Huntjens et al. likewise reported lower subsequent fracture risk with FLS care, although Huntjens et al. observed a weaker estimate at 12 than at 24 months (25, 26). By contrast, a population-based natural experiment across English hospitals found lower mortality after FLS introduction but no clear reduction in second hip fracture (27). Heterogeneity in index fracture type, service intensity, treatment uptake, follow-up duration, and outcome definition may explain these differences. Our findings extend this literature to an older vertebral fracture population and to a pathway explicitly coupled with zoledronic acid; they do not establish a universal effect across FLS models.

The FLS literature describes coordinator-based systems connecting orthopedic teams with osteoporosis and falls services, patients, and primary care to support identification, assessment, education, treatment, and monitoring (28). Contemporary multidisciplinary models likewise position FLS as the process that moves patients from fracture recognition to an individualized secondary prevention plan (29). In this registry, however, the binary FLS field does not establish which components were delivered as part of FLS or how consistently they were implemented. This limits comparison with a fully characterized service. Continuity after discharge may also be disrupted by role ambiguity, communication gaps, and incomplete primary-care follow-up (30). A pharmacologic contribution is plausible: randomized evidence in women aged 65 years or older showed that intermittent zoledronate reduced vertebral and non-vertebral fragility fractures (31). Our positive discharge ZOL field did not verify infusion, repeat dosing, or adherence. The combined exposure and small component-only groups therefore cannot separate service effects, drug effects, or their interaction.

We used the extended source cohort to describe a broader range of recorded component patterns, not to attribute the primary 730-day association to either component. Component-only patterns numbered 10 in the primary source cohort and 44 in the extended source cohort. Their greater frequency after including 2022 admissions may reflect evolving prescribing practice and efforts to close the treatment gap outside formal FLS care. Calendar adjustment cannot fully capture changes in service organization, treatment selection, or outcome recording. These possibilities further limit causal interpretation of the component counts.

The present analysis compared one recorded combined pathway with a comparator pathway and did not evaluate FLS alone, antiresorptive therapy alone, or an integrated hospital-to-community process as alternative strategies. It therefore does not establish superiority of one secondary prevention strategy over another. The recorded combined pathway warrants further evaluation in studies with component-level exposure data and adequate outcome information.

Residual treatment selection bias remains the principal limitation. Recorded ZOL prescribing may depend on clinical stability, renal treatment eligibility, prognosis and life expectancy, frailty, cognition, functional status and mobility, contraindications or intolerance to alternative therapies, patient preference, and ability to attend follow-up, which the registry did not adequately measure. Weighting addresses only recorded variables. Outcome information was also limited to 37 refractures and 26 competing deaths; regularization, multiple imputation, and bootstrap estimation do not add events or remove uncertainty from sparse data. Reduced model and single-event influence analyses were informative but cannot establish the absence of bias. Multiple imputation assumes that its included predictors sufficiently explain missingness; the unknown reasons for absent blood pressure measurements mean that missing-not-at-random bias remains possible. Anatomical ICD codes, the OP field, and incompletely characterized bone density measures did not independently validate osteoporotic etiology. Fracture etiology and its documentation may influence both treatment selection and subsequent fracture risk; the direction of any resulting bias is uncertain. Outcomes outside the linked systems and uncertain treatment administration are additional potential sources of misclassification.

FLS timing was not independently established, and some adjusted variables may have been recorded after pathway activity. Temporal misclassification and adjustment for downstream care-related measurements therefore remain possible despite the admission record sensitivity analysis. The dated ZOL restriction excluded 20 pathway patients, including 4 of the 8 pathway refractures. Record completeness may be associated with patient characteristics, care processes, or outcome recording. The larger association in this subset should not be interpreted as stronger evidence of treatment efficacy or confirmation of infusion.

External validity is limited. The cohort consisted of adults aged 65 years or older with strictly coded thoracic or lumbar vertebral fractures who were hospitalized, underwent surgery, and were represented in a single regional registry in China. Vertebral augmentation accounted for 97.4% of pathway procedures and 94.4% of comparator procedures. The findings therefore do not directly apply to conservatively managed or incidentally detected vertebral fractures, patients too frail to undergo an invasive procedure, other healthcare systems, or FLS programs with different organizational and follow-up structures.

## Conclusions

Among older surgically treated vertebral fracture inpatients with overlapping measured characteristics, a recorded FLS indicator plus a positive discharge ZOL prescription/dispensing field was associated with a lower estimated 730-day refracture incidence than a pathway with neither recorded FLS nor a positive discharge bisphosphonate field. This observational, pathway-level association does not establish confirmed intravenous administration or the independent effect of FLS or zoledronic acid. Furthermore, these findings may lack generalizability to conservatively managed or incidentally detected vertebral fractures, very frail non-surgical patients, other healthcare systems, or differently organized FLS programs. The small number of events and unmeasured treatment selection factors warrant cautious interpretation.

## Data Availability

Publicly available datasets were analyzed in this study. These data can be found here: The datasets analyzed in this study are publicly available through Figshare at https://doi.org/10.6084/m9.figshare.30971476 and are described in the associated Scientific Data article.
