# Supplementary material for: Recorded fracture liaison service plus discharge zoledronic acid prescription and 730-day refracture risk after surgically treated vertebral fracture in older adults: a target-trial-informed discharge landmark analysis
Source: Front Med (Lausanne). 2026 Oct 14;13:1972395. doi: 10.3389/fmed.2026.1972395 (PMC13713360; doi:10.3389/fmed.2026.1972395)
Supplement: Supplementary file 1 [file Datasheet1.docx]

**Table S1. Exposure, eligibility and outcome operationalization.**

| **Construct** | **Released field and analytic rule** | **Interpretation** |
| --- | --- | --- |
| Recorded FLS | FLS > 0 | Binary recorded use; no component-level implementation or service date. |
| Discharge zoledronic acid | ZOL > 0 | Positive discharge prescription/dispensing field; not verified intravenous administration. |
| Pathway | FLS > 0 and ZOL > 0 | Recorded combined classification; FLS timing is not independently verified. ALN co-prescription does not exclude it. |
| Comparator | FLS = 0, ZOL = 0 and ALN = 0 | Neither recorded FLS nor a positive discharge zoledronic-acid/alendronate field. |
| Dated ZOL restriction | Non-missing RYQzoltime between admission and discharge for pathway patients | A dated record only; the dictionary label does not align with observed dates. Comparator patients are retained. |
| Index fracture | ICD begins S22.0 or S32.0 | Thoracic or lumbar vertebral-fracture anatomy, not independent etiologic validation. |
| Procedure family | SURGERY 7/8: augmentation; 9/10: fixation/fusion | Recorded index procedure, not a verified admission-baseline variable. |
| First refracture | Refracture > 0 and first event within horizon, before or on death date | Recorded hospitalized subsequent fracture; same-date events classified as refracture. |
| Competing death | Death before first recorded refracture, within horizon | Death retained as a competing event for the primary Aalen-Johansen estimate. |

FLS, fracture liaison service; ZOL, zoledronic acid; ALN, alendronate. The public release does not provide a component-specific FLS protocol, service date, case-identification procedures, falls assessment, patient education, post-discharge FLS contacts, or coordination with primary care. Osteoporosis, bone-density, laboratory, and medication fields provide clinical documentation but do not verify that these assessments or treatments were delivered through FLS. Positive medication fields indicate discharge prescription/dispensing records and do not verify administration, repeat dosing, or adherence. The original source registry concerns osteoporotic fractures, but this secondary analysis cannot independently validate fracture etiology or administered treatment.

**Table S2. Discharge-landmark study specification.**

| **Element** | **Specification** |
| --- | --- |
| Population | Patients aged >=65 years with recorded surgery for thoracic/lumbar vertebral fracture in the released registry. |
| Primary enrollment | Admissions during 2019-2021; valid admission-operation-discharge chronology; alive and refracture-free at discharge. |
| Time zero | Index discharge. ZOL is a discharge field; treating recorded FLS as an index-care attribute is an operational assumption because no independent FLS timestamp is available. Whether recorded FLS use preceded discharge and whether all propensity-score covariates preceded pathway activity cannot be independently verified. |
| Comparison | Recorded FLS plus positive discharge ZOL versus FLS = ZOL = ALN = 0. Single-component patterns excluded from binary inference. |
| Outcome | First recorded hospitalized refracture; death before refracture is competing. |
| Follow-up | 730 days for primary analysis; fixed refracture-ascertainment cutoff 24 March 2024. |
| Estimand | Adjusted pathway-level association in the propensity-score overlap population, expressed as cumulative-incidence RD and RR. |
| Analysis | MI50, ridge PS (C = 1), overlap weighting, Aalen-Johansen estimation; 1,000 patient-level bootstraps. |
| Extended analysis | 2019-2022 admissions, 365 days, N = 770. Different calendar cohort and horizon, not a longitudinal comparison of treatment onset. |

This is a target-trial-informed comparison of recorded pathway states among discharge survivors, not randomized assignment, a new-user trial, or verified treatment administration.

**Table S3. Candidate phenotype screens in the primary cohort.**

| **Screen** | **Retained N** | **Pathway/ comparator** | **Refractures** | **Competing deaths** |
| --- | --- | --- | --- | --- |
| Locked anatomical cohort | 585 | 266/319 | 37 | 26 |
| Require released M80 code | 0 | 0/0 | 0 | 0 |
| Exclude released M84 codes | 585 | 266/319 | 37 | 26 |
| Require OP = 1 | 212 | 130/82 | 17 | 8 |
| OP = 1 and no recorded cancer history | 209 | 128/81 | 17 | 8 |
| Require BMD measurement flag | 295 | 167/128 | 17 | 14 |
| Positive standard-site density available | 295 | 167/128 | 17 | 14 |
| OP = 1 and positive density available | 153 | 96/57 | 12 | 4 |
| No recorded cancer history | 579 | 263/316 | 37 | 25 |

These are documentation screens, not validated diagnostic criteria or effect analyses. No M80/M84 codes were present in the released ICD field; absence cannot establish that the underlying conditions were absent. M80 denotes osteoporosis with pathological fracture; pathological fracture is not synonymous with malignant fracture. OP = 1 retains 212 patients but may reflect differential documentation. Site-specific density fields lack sufficient diagnostic metadata for an independent validated osteoporosis definition. Primary eligibility was not changed.

**Table S4. Exact propensity-score specifications.**

| **Model** | **Terms** | **Encoded terms** |
| --- | --- | --- |
| Primary model A | 29 | age; sex_male; year_2020; year_2021; quarter_2; quarter_3; quarter_4; thoracic; fixation; smoker; drink; sbp; dbp; bmi; hemoglobin; albumin; creatinine; cci; hypertension; diabetes; heart; lung; kidney; brain; tumor; urea_nitrogen; calcium; neutrophil; lymphocyte |
| Disease-domain PS without CCI | 25 | age; sex_male; year_2020; year_2021; quarter_2; quarter_3; quarter_4; thoracic; fixation; smoker; drink; sbp; dbp; bmi; hemoglobin; albumin; creatinine; hypertension; domain_diabetes; domain_cardiovascular; domain_pulmonary; domain_renal; domain_neurologic; domain_malignancy; domain_liver |
| CCI plus selected clinical indicators | 20 | age; sex_male; year_2020; year_2021; quarter_2; quarter_3; quarter_4; thoracic; fixation; smoker; drink; sbp; dbp; bmi; hemoglobin; albumin; creatinine; cci; hypertension; domain_renal |
| Clinical-core PS (11 terms) | 11 | age; sex_male; year_2020; year_2021; thoracic; cci; domain_renal; bmi; albumin; creatinine; calcium |
| Admission-record reduced PS | 20 | age; sex_male; year_2020; year_2021; quarter_2; quarter_3; quarter_4; thoracic; smoker; drink; sbp; dbp; cci; hypertension; diabetes; heart; lung; kidney; brain; tumor |
| Primary PS without CCI | 28 | age; sex_male; year_2020; year_2021; quarter_2; quarter_3; quarter_4; thoracic; fixation; smoker; drink; sbp; dbp; bmi; hemoglobin; albumin; creatinine; hypertension; diabetes; heart; lung; kidney; brain; tumor; urea_nitrogen; calcium; neutrophil; lymphocyte |
| Primary PS without urea nitrogen | 28 | age; sex_male; year_2020; year_2021; quarter_2; quarter_3; quarter_4; thoracic; fixation; smoker; drink; sbp; dbp; bmi; hemoglobin; albumin; creatinine; cci; hypertension; diabetes; heart; lung; kidney; brain; tumor; calcium; neutrophil; lymphocyte |

Intercepts are not counted. Full clinical labels for primary terms are in Table 1 and Supplementary Table S6. Year and quarter reference categories are 2019 and quarter 1; thoracic is versus lumbar; fixation/fusion is versus augmentation. Disease domains combine the relevant recorded general-history and Charlson component flags (any positive). The renal domain combines kidney and CKD. Admission-record models omit procedure, BMI, ASA and laboratory terms from both PS and MI predictors; no independent admission timestamp is asserted for every retained history. CCI/urea deletion sensitivities retain the full MI auxiliary pool. MI predictors additionally include exposure, event indicators, log(1 + follow-up time), and exposure-event interactions. The primary MI PS has no missingness indicators; the median-imputation sensitivity adds five independent missingness-pattern indicators.

**Table S5. Missing covariate values before imputation.**

| **Variable** | **Pathway, n (%)** | **Comparator, n (%)** | **Overall, n (%)** |
| --- | --- | --- | --- |
| Systolic blood pressure, mmHg | 66 (24.8) | 94 (29.5) | 160 (27.4) |
| Diastolic blood pressure, mmHg | 66 (24.8) | 94 (29.5) | 160 (27.4) |
| Body mass index, kg/m2 | 12 (4.5) | 55 (17.2) | 67 (11.5) |
| Hemoglobin, g/L | 3 (1.1) | 6 (1.9) | 9 (1.5) |
| Albumin, g/L | 5 (1.9) | 7 (2.2) | 12 (2.1) |
| Creatinine, umol/L | 5 (1.9) | 6 (1.9) | 11 (1.9) |
| Urea nitrogen, mmol/L | 5 (1.9) | 6 (1.9) | 11 (1.9) |
| Calcium, mmol/L | 5 (1.9) | 6 (1.9) | 11 (1.9) |
| Neutrophils, 10^9/L | 3 (1.1) | 6 (1.9) | 9 (1.5) |
| Lymphocytes, 10^9/L | 3 (1.1) | 6 (1.9) | 9 (1.5) |

Denominators are 266, 319 and 585. Other primary propensity-score covariates were complete. SBP and DBP were missing in the same 160 patients. Missingness reasons were not released. Multiple imputation was used in the primary analysis; the primary propensity-score model contained 29 clinical terms and no missingness-indicator covariates. In the median-imputation sensitivity analysis, five independent missingness-pattern indicators were retained: SBP/DBP; hemoglobin/neutrophils/lymphocytes; creatinine/urea nitrogen/calcium; BMI; and albumin. Complete-case analysis was also performed as a sensitivity analysis. Observed missingness patterns do not establish the missing-at-random assumption.

**Table S6. Balance of the fitted propensity terms and common clinical panel.**

| **Covariate** | **In primary PS** | **Before median \|SMD\|** | **After median \|SMD\|** | **After maximum \|SMD\|** |
| --- | --- | --- | --- | --- |
| Age, years | Yes | 0.128 | 0.000 | 0.000 |
| Male sex | Yes | 0.271 | 0.003 | 0.004 |
| Charlson Comorbidity Index | Yes | 0.131 | 0.006 | 0.006 |
| Hypertension history | Yes | 0.202 | 0.003 | 0.004 |
| Diabetes history | Yes | 0.047 | 0.001 | 0.001 |
| Heart disease history | Yes | 0.007 | 0.001 | 0.001 |
| Pulmonary disease history | Yes | 0.012 | 0.001 | 0.001 |
| Kidney disease history | Yes | 0.143 | 0.002 | 0.002 |
| Brain/cerebrovascular history | Yes | 0.052 | 0.001 | 0.001 |
| Malignancy history | Yes | 0.019 | 0.002 | 0.002 |
| Smoking history | Yes | 0.043 | 0.001 | 0.002 |
| Alcohol-consumption history | Yes | 0.128 | 0.001 | 0.001 |
| Systolic blood pressure, mmHg | Yes | 0.110 | 0.001 | 0.002 |
| Diastolic blood pressure, mmHg | Yes | 0.072 | 0.001 | 0.002 |
| Body mass index, kg/m2 | Yes | 0.025 | 0.000 | 0.001 |
| Hemoglobin, g/L | Yes | 0.100 | 0.001 | 0.001 |
| Albumin, g/L | Yes | 0.075 | 0.001 | 0.002 |
| Creatinine, umol/L | Yes | 0.109 | 0.003 | 0.003 |
| Urea nitrogen, mmol/L | Yes | 0.007 | 0.003 | 0.003 |
| Calcium, mmol/L | Yes | 0.031 | 0.002 | 0.002 |
| Neutrophils, 10^9/L | Yes | 0.177 | 0.002 | 0.002 |
| Lymphocytes, 10^9/L | Yes | 0.077 | 0.001 | 0.001 |
| Admission in 2020 | Yes | 0.628 | 0.040 | 0.041 |
| Admission in 2021 | Yes | 0.004 | 0.021 | 0.021 |
| Admission quarter 2 | Yes | 0.086 | 0.010 | 0.011 |
| Admission quarter 3 | Yes | 0.275 | 0.022 | 0.023 |
| Admission quarter 4 | Yes | 0.050 | 0.014 | 0.014 |
| Thoracic vertebral fracture | Yes | 0.047 | 0.003 | 0.004 |
| Fixation or fusion | Yes | 0.152 | 0.045 | 0.046 |
| Diabetes domain | No | 0.047 | 0.001 | 0.001 |
| Cardiovascular domain | No | 0.067 | 0.021 | 0.026 |
| Pulmonary domain | No | 0.012 | 0.001 | 0.001 |
| Renal domain | No | 0.143 | 0.002 | 0.002 |
| Neurologic domain | No | 0.052 | 0.001 | 0.001 |
| Malignancy domain | No | 0.019 | 0.002 | 0.002 |
| Liver disease domain | No | 0.112 | 0.011 | 0.013 |
| Myocardial infarction (CCI item) | No | 0.079 | 0.007 | 0.009 |
| Congestive heart failure (CCI item) | No | 0.196 | 0.066 | 0.077 |
| Peripheral vascular disease (CCI item) | No | 0.162 | 0.042 | 0.052 |
| Dementia (CCI item) | No | 0.000 | 0.000 | 0.000 |
| Cerebrovascular disease (CCI item) | No | 0.094 | 0.029 | 0.033 |
| Connective tissue disease (CCI item) | No | 0.011 | 0.042 | 0.048 |
| Peptic ulcer disease (CCI item) | No | 0.011 | 0.019 | 0.027 |
| Mild liver disease (CCI item) | No | 0.000 | 0.000 | 0.000 |
| Uncomplicated diabetes (CCI item) | No | 0.047 | 0.001 | 0.001 |
| Chronic lung disease (CCI item) | No | 0.011 | 0.078 | 0.087 |
| Hemiplegia (CCI item) | No | 0.000 | 0.000 | 0.000 |
| Chronic kidney disease (CCI item) | No | 0.112 | 0.006 | 0.008 |
| Complicated diabetes (CCI item) | No | 0.000 | 0.000 | 0.000 |
| Solid tumor (CCI item) | No | 0.019 | 0.002 | 0.002 |
| Leukemia (CCI item) | No | 0.000 | 0.000 | 0.000 |
| Malignant lymphoma (CCI item) | No | 0.000 | 0.000 | 0.000 |
| Moderate/severe liver disease (CCI item) | No | 0.112 | 0.011 | 0.013 |
| Metastatic malignancy (CCI item) | No | 0.000 | 0.000 | 0.000 |
| AIDS (CCI item) | No | 0.000 | 0.000 | 0.000 |

Each SMD uses the corresponding completed dataset and its own overlap weights. The denominator is the unweighted pooled within-group SD. Medians/maxima summarize 50 completed datasets, not averaged covariates. Constant clinical indicators contribute zero imbalance and do not enter PS fitting. Complete-case and admission-restricted panels differ and must not be equated to full-panel balance. CCI items and general recorded histories are distinct released fields.

**Table S7. Primary propensity-score and weighting diagnostics.**

| **Diagnostic** | **Value** |
| --- | --- |
| Cohort and groups | 585; pathway 266, comparator 319 |
| Non-intercept PS terms | 29 |
| Design matrix rank across imputations | 29 to 29 |
| Maximum VIF across imputations | 4.29 |
| Total ESS, median (range) | 453.2 (449.0 to 455.2) |
| Pathway ESS, median | 214.9 |
| Comparator ESS, median | 239.5 |
| PS range across patients and imputations | 0.0035 to 0.9806 |
| Overlap weight range across patients and imputations | 0.0035 to 0.9299 |
| Maximum absolute SMD, fitted terms | 0.046 |
| Maximum absolute SMD, common clinical panel | 0.087 |
| Complete 730-day outcome opportunity | 585/585; no event-free administrative censoring before 730 days |
| Imputations / patient bootstrap samples | 50 / 1,000 |
| Failed bootstrap samples | 0 |

ESS, effective sample size; VIF, variance inflation factor. Ranges and maxima refer to all 50 completed datasets unless otherwise stated. Full rank and finite VIF do not establish absence of confounding. Overlap weighting used all 585 patients; common-support exclusion was a separate sensitivity analysis.

**Table S8. Complete primary and sensitivity estimates.**

| **Analysis** | **N (P/C) Events R/D** | **Risks, % (95% CI)** | **Contrasts (95% CI)** |
| --- | --- | --- | --- |
| Primary model A | 585 (266/319) 37/26 | P: 2.40 (0.76 to 4.31) C: 7.02 (4.12 to 10.29) | RD: -4.63 (-8.38 to -1.24) RR: 0.34 (0.10 to 0.76) |
| Disease-domain PS without CCI | 585 (266/319) 37/26 | P: 2.35 (0.77 to 4.29) C: 7.35 (4.49 to 10.60) | RD: -5.00 (-8.49 to -1.58) RR: 0.32 (0.10 to 0.70) |
| CCI plus selected clinical indicators | 585 (266/319) 37/26 | P: 2.38 (0.80 to 4.32) C: 7.37 (4.47 to 10.73) | RD: -4.99 (-8.59 to -1.55) RR: 0.32 (0.11 to 0.69) |
| Clinical-core PS (11 terms) | 585 (266/319) 37/26 | P: 2.99 (1.03 to 5.21) C: 7.54 (4.74 to 10.50) | RD: -4.54 (-7.75 to -0.90) RR: 0.40 (0.14 to 0.82) |
| Admission-record reduced PS | 585 (266/319) 37/26 | P: 2.39 (0.73 to 4.28) C: 7.03 (4.28 to 10.28) | RD: -4.65 (-8.28 to -1.45) RR: 0.34 (0.11 to 0.72) |
| Primary PS without CCI | 585 (266/319) 37/26 | P: 2.44 (0.81 to 4.41) C: 7.36 (4.45 to 10.56) | RD: -4.92 (-8.61 to -1.39) RR: 0.33 (0.10 to 0.72) |
| Primary PS without urea nitrogen | 585 (266/319) 37/26 | P: 2.45 (0.79 to 4.41) C: 7.19 (4.21 to 10.55) | RD: -4.74 (-8.46 to -1.34) RR: 0.34 (0.11 to 0.75) |
| Median imputation + independent patterns | 585 (266/319) 37/26 | P: 2.18 (0.70 to 4.06) C: 6.91 (4.05 to 10.09) | RD: -4.73 (-8.40 to -1.56) RR: 0.32 (0.10 to 0.69) |
| Complete cases | 392 (194/198) 23/15 | P: 1.68 (0.16 to 3.97) C: 6.19 (2.65 to 9.84) | RD: -4.51 (-8.68 to -0.50) RR: 0.27 (0.02 to 0.85) |
| Dated ZOL-record restriction | 565 (246/319) 33/25 | P: 0.77 (0.09 to 1.77) C: 6.88 (3.83 to 10.50) | RD: -6.11 (-9.82 to -2.69) RR: 0.11 (0.01 to 0.33) |
| PKP-only restriction | 547 (255/292) 37/26 | P: 2.52 (0.62 to 4.82) C: 7.53 (4.38 to 10.99) | RD: -5.01 (-9.07 to -1.08) RR: 0.33 (0.08 to 0.77) |
| Common-support restriction | 536 (262/274) 29/21 | P: 2.44 (0.72 to 4.38) C: 6.91 (4.05 to 10.33) | RD: -4.47 (-8.44 to -1.29) RR: 0.35 (0.10 to 0.76) |
| Primary PS plus ASA class >=3 | 585 (266/319) 37/26 | P: 2.39 (0.78 to 4.31) C: 7.01 (4.13 to 10.22) | RD: -4.62 (-8.31 to -1.28) RR: 0.34 (0.10 to 0.75) |
| Death-censored net risk (730 days) | 585 (266/319) 37/26 | P: 2.46 (0.77 to 4.40) C: 7.10 (4.15 to 10.39) | RD: -4.64 (-8.42 to -1.17) RR: 0.35 (0.11 to 0.77) |
| Extended 2019-2022 cohort (365 days) | 770 (324/446) 22/17 | P: 1.78 (0.30 to 3.61) C: 3.02 (1.41 to 4.89) | RD: -1.24 (-3.62 to 1.07) RR: 0.59 (0.09 to 1.56) |

P/C, pathway/comparator; R/D, refractures/deaths before refracture. RD is in percentage points. Each row uses 1,000 patient-level bootstrap samples. All rows use 730 days except the extended 2019-2022 row (365 days). Death-censored net risk is 1 minus Kaplan-Meier survival and differs from competing-risk cumulative incidence. Remaining rows use Aalen-Johansen. These are specification or population sensitivity analyses, not independent confirmatory efficacy discoveries.

**Table S9. Design diagnostics across all inferential analyses.**

| **Analysis** | **PS terms** | **ESS median** | **Maximum \|SMD\| fitted** | **Maximum \|SMD\| common panel** |
| --- | --- | --- | --- | --- |
| Primary model A | 29 | 453.2 | 0.046 | 0.087 |
| Disease-domain PS without CCI | 25 | 467.3 | 0.040 | 0.265 |
| CCI plus selected clinical indicators | 20 | 466.9 | 0.042 | 0.268 |
| Clinical-core PS (11 terms) | 11 | 494.0 | 0.033 | 0.322 |
| Admission-record reduced PS | 20 | 469.5 | 0.037 | Not evaluated* |
| Primary PS without CCI | 28 | 458.7 | 0.044 | 0.185 |
| Primary PS without urea nitrogen | 28 | 456.7 | 0.043 | 0.178 |
| Median imputation + independent patterns | 34 | 445.4 | 0.046 | 0.078 |
| Complete cases | 29 | 311.6 | 0.062 | 0.179 |
| Dated ZOL-record restriction | 29 | 421.9 | 0.053 | 0.087 |
| PKP-only restriction | 28 | 414.8 | 0.047 | 0.083 |
| Common-support restriction | 29 | 451.9 | 0.045 | 0.096 |
| Primary PS plus ASA class >=3 | 30 | 453.1 | 0.046 | 0.088 |
| Death-censored net risk (730 days) | 29 | 453.2 | 0.046 | 0.087 |
| Extended 2019-2022 cohort (365 days) | 31 | 613.5 | 0.042 | 0.072 |

Maxima are the worst across completed datasets. *Admission-record analysis evaluates its retained admission panel only (maximum 0.037), not the full clinical panel. The median-imputation sensitivity has 29 clinical terms plus five independent missingness indicators. Constant terms are omitted within the corresponding fit. All analyses have 1,000 completed bootstrap samples with no failed samples; one common-support bootstrap draw had a rank-deficient design in at least one imputation, was retained, and was independently verified. The primary matrix and the two single-term deletion models were full-rank in all analyzed bootstrap draws. Maximum VIF: primary 4.29; without CCI 2.13; without urea nitrogen 4.25.

**Table S10. Event information in date-record and complete-case subsets.**

| **Subset** | **N** | **Refractures** | **Competing deaths** |
| --- | --- | --- | --- |
| Pathway with dated ZOL record | 246 | 4 | 11 |
| Pathway without dated ZOL record | 20 | 4 | 1 |
| Comparator | 319 | 29 | 14 |
| Complete cases - comparator | 198 | 18 | 7 |
| Complete cases - pathway | 194 | 5 | 8 |
| Excluded from complete cases - comparator | 121 | 11 | 7 |
| Excluded from complete cases - pathway | 72 | 3 | 4 |

All counts use 730 days. Dated-record status does not confirm infusion. Single-event influence analysis omitted each of the 37 refracture patients in turn, re-imputed covariates and refitted the primary model; RD ranged from -5.19 to -3.87 percentage points. These are influence diagnostics without new confidence intervals; no patient was removed from the primary analysis.

**Table S11. Potential follow-up and actual early administrative censoring.**

| **Year** | **Group** | **N** | **Potentially complete 365 days** | **Potentially complete 730 days** | **Event-free early censoring at 730 days** |
| --- | --- | --- | --- | --- | --- |
| 2019 | Pathway | 42 | 42 (100.0%) | 42 (100.0%) | 0 |
| 2019 | Comparator | 143 | 143 (100.0%) | 143 (100.0%) | 0 |
| 2020 | Pathway | 136 | 136 (100.0%) | 136 (100.0%) | 0 |
| 2020 | Comparator | 71 | 71 (100.0%) | 71 (100.0%) | 0 |
| 2021 | Pathway | 88 | 88 (100.0%) | 88 (100.0%) | 0 |
| 2021 | Comparator | 105 | 105 (100.0%) | 105 (100.0%) | 0 |
| 2022 | Pathway | 58 | 58 (100.0%) | 22 (37.9%) | 35 |
| 2022 | Comparator | 127 | 127 (100.0%) | 19 (15.0%) | 102 |

Potential completeness is based on discharge date and the fixed ascertainment cutoff, regardless of subsequent events. Early administrative censoring counts only patients without refracture or competing death before their available follow-up ends. The 2022 subgroup has 35 + 102 = 137 such censoring events. The primary 2019-2021 cohort is fully mature at 730 days. The extended inferential analysis uses 365 days; no extended 730-day effect is reported.

**Table S12. Recorded component patterns in the extended source cohort.**

| **Pattern** | **N** | **Refractures by 730 days** | **Competing deaths by 730 days** | **Positive ZOL/ALN** |
| --- | --- | --- | --- | --- |
| Neither component | 446 | 32 | 19 | 0/0 |
| FLS only | 14 | 2 | 1 | 0/0 |
| Bisphosphonate only | 30 | 1 | 6 | 22/8 |
| Recorded FLS plus positive discharge ZOL | 324 | 10 | 13 | 324/5 |

N = 814 at the discharge landmark, including 44 component-only patients excluded from the extended binary cohort of 770. Counts are descriptive and unadjusted, with incomplete 730-day follow-up for some 2022 discharges; they are not comparable cumulative risks or independent component effects. Positive ZOL/ALN fields denote prescription/dispensing records.

**Table S13. Recorded supportive discharge medication patterns.**

| **Pattern** | **N** |
| --- | --- |
| Strict calcium or vitamin D without bisphosphonate or calcitonin | 293 |
| Calcitonin only without calcium, vitamin D, or bisphosphonate | 12 |
| Any calcitonin | 213 |
| Any calcium | 338 |
| Any vitamin D | 673 |

Denominator: 814 patients in the extended discharge-landmark source cohort. The first two rows are mutually restricted patterns; the remaining any-use rows overlap and must not be summed. Positive discharge fields indicate recorded prescribing/dispensing, not verified administration or adherence.

**Figure S1. Propensity-score distributions in the primary cohort.** Group-specific density histograms are averaged across 50 imputed datasets using common 0.05-wide bins. Each imputation uses its own fitted primary propensity model. Densities describe patients before overlap weighting; stacked imputed records are not treated as independent patients.

**Figure S2. Balance of the 29 primary propensity-score terms.** Before-weighting points are median absolute SMDs across 50 completed datasets. After-weighting points are the maximum absolute SMD across the same datasets, making the displayed residual imbalance conservative across imputations. The dashed line is 0.10. This figure covers fitted terms; Supplementary Table S6 also includes the wider common clinical panel. The maximum after-weighting SMD was 0.046 for fitted terms and 0.087 for the common panel.

**Figure S3. Residual imbalance under alternative propensity specifications.** Each marker is the largest absolute weighted SMD over all variables in the indicated panel and all 50 completed datasets. Simplified models balanced their fitted terms but left larger residual differences in the common clinical panel. These diagnostics accompany specification sensitivity analyses; they do not rank models by statistical significance.

**Figure S4. Leave-one-refracture-patient influence on the primary estimate.** Each point omits one of the 37 patients with a refracture, repeats multiple imputation and primary-model estimation, and shows the resulting 730-day risk difference. The dashed line is the full-cohort estimate. Omission estimates ranged from -5.19 to -3.87 percentage points. This is a descriptive influence diagnostic without confidence intervals; no patient was excluded from the primary analysis.

**Figure S5. Potential follow-up completeness by admission year.** (A) Potentially complete 365-day and (B) 730-day refracture ascertainment before 24 March 2024, based on discharge date rather than observed event status. In 2022, 22/58 pathway and 19/127 comparator patients could potentially reach 730 days. All 770 patients in the extended binary cohort could potentially reach 365 days. Actual early administrative censoring is reported separately in Supplementary Table S11.
